# Post-translational Modifications in Regulation of Chloroplast Function: Recent Advances

**DOI:** 10.3389/fpls.2017.00240

**Published:** 2017-02-23

**Authors:** Magda Grabsztunowicz, Minna M. Koskela, Paula Mulo

**Affiliations:** Molecular Plant Biology, Department of Biochemistry, University of TurkuTurku, Finland

**Keywords:** chloroplast, phosphorylation, photosynthesis, post-translational modification, redox regulation

## Abstract

Post-translational modifications (PTMs) of proteins enable fast modulation of protein function in response to metabolic and environmental changes. Phosphorylation is known to play a major role in regulating distribution of light energy between the Photosystems (PS) I and II (state transitions) and in PSII repair cycle. In addition, thioredoxin-mediated redox regulation of Calvin cycle enzymes has been shown to determine the efficiency of carbon assimilation. Besides these well characterized modifications, recent methodological progress has enabled identification of numerous other types of PTMs in various plant compartments, including chloroplasts. To date, at least N-terminal and Lys acetylation, Lys methylation, Tyr nitration and S-nitrosylation, glutathionylation, sumoylation and glycosylation of chloroplast proteins have been described. These modifications impact DNA replication, control transcriptional efficiency, regulate translational machinery and affect metabolic activities within the chloroplast. Moreover, light reactions of photosynthesis as well as carbon assimilation are regulated at multiple levels by a number of PTMs. It is likely that future studies will reveal new metabolic pathways to be regulated by PTMs as well as detailed molecular mechanisms of PTM-mediated regulation.

## Introduction

Chloroplasts are sites of versatile metabolism. In addition to photosynthetic reactions, chloroplasts host a number of other processes, such as nitrogen and sulfur assimilation, amino acid and fatty acid biosynthesis as well as accumulation of pigments, photoreceptors, and hormones. Chloroplasts are surrounded by the envelope membrane, and the majority of nuclear-encoded chloroplast proteins are imported through the envelope into the plastid via the Toc/Tic machinery. The subchloroplastic destination of a specific protein is determined by the information buried within the primary amino acid sequence, either in the form of cleavable transit peptide or as an internal targeting signal. Due to their endosymbiotic origin, biosynthesis and function of chloroplasts is not only dependent on nuclear control, but also on the expression of approximately 120 plastome encoded genes, mostly involved in photosynthesis and plastid gene expression (Sugiura, 1992; Green, 2011). Obviously, coordination of gene expression between these compartments as well as integration of plastid metabolism with the rest of the cell are required to induce appropriate physiological responses to various environmental stimuli, thereby enabling successful growth and reproduction of the plants. This coordination takes place at many different levels, including the control of nuclear and plastid transcription, RNA processing and translation, protein translocation and assembly of protein complexes as well as functional adjustments of specific enzymes and/or pathways.

**FIGURE 1:**
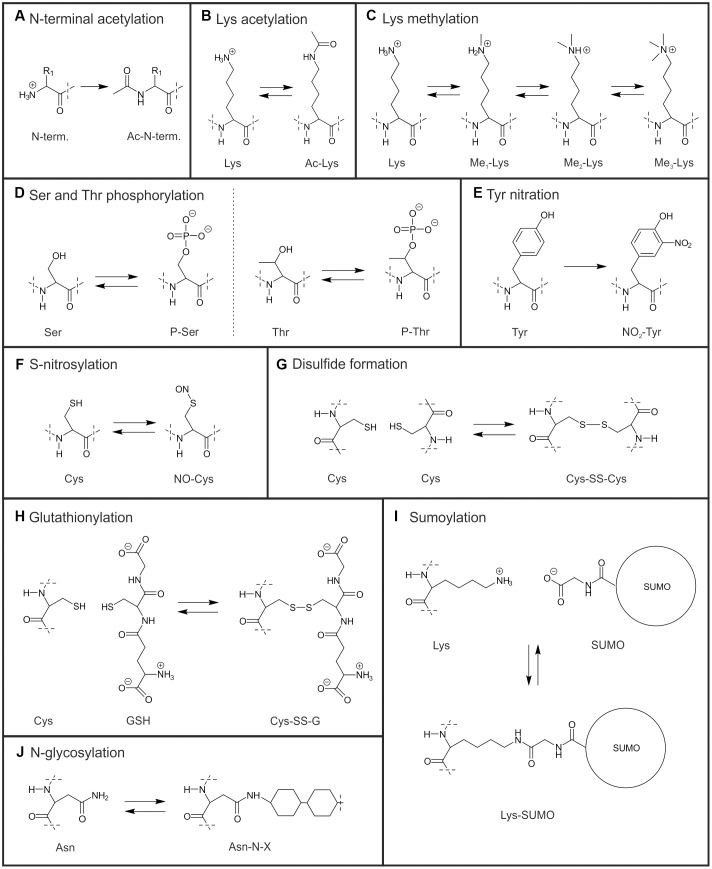
**Molecular structures of chloroplast Post-translational modifications (PTMs). (A)** N-terminal acetylation (Ac denotes acetyl group and N-term. the N-terminal amino acid of a protein). **(B)** Lys acetylation. **(C)** Lys mono-, di- and trimethylation (Me denotes methyl group). **(D)** Ser and Thr phosphorylation (P denotes phospho group). **(E)** Tyr nitration. **(F)** S-nitrosylation. **(G)** Disulfide formation (-SS- denotes disulfide bridge). **(H)** Glutathionylation of Cys (GSH denotes reduced and G oxidized glutathione). **(I)** Lys sumoylation. **(J)** N-glycosylation of Asn (-N-X denotes N-linked glycosyl group). Dash line indicates where structures have been cut off.

Recent interest and methodological progress on PTMs of non-histone proteins has revealed that also a great number of chloroplast proteins are post-translationally modified, which denotes for covalent processing of a mature protein. The most well-studied chloroplast protein modifications: (de)phosphorylation, conveyed by kinases and phosphatases, and oxidation-reduction (including disulfide-thiol exchange of Cys residues, regulated via thioredoxins) have been extensively reviewed (e.g., Buchanan and Balmer, 2005; Tikkanen and Aro, 2012; Michelet et al., 2013; Rochaix, 2013) and thus are not described in detail in the present article. Other PTM types, such as acetylation, methylation, glycosylation, nitration and nitrosylation, sumoylation, and glutathionylation have been identified in chloroplast proteins much later. As only limited information is available for these PTMs, it is currently not possible to conclude whether a given PTM is found in the chloroplasts of all plant and algal species, or whether it is specific for a certain group of organisms. In addition to the PTMs modifying a given amino acid, recent studies have shown that a number of chloroplast proteins are prone to N-terminal trimming resulting in different N-termini or N-terminal acetylation (Lehtimäki et al., 2015; Rowland et al., 2015). It is intriguing that both nuclear- and chloroplast-encoded proteins may be subjected to these modifications (Lehtimäki et al., 2015). In most cases the site of the PTM (cytosol or plastid) and/or the responsible enzymes have remained obscure. PTMs alter the physicochemical properties and thus the function of proteins in different ways depending on the modification and the molecular environment. The molecular structures of the different chloroplast PTM are presented in **Figure 1**. Here, we will draw together the current understanding of the PTMs regulating distinct metabolic processes in chloroplasts, and review the known physiological effects of these modifications.

## Chloroplast Machinery for DNA Replication and Gene Expression

**FIGURE 2:**
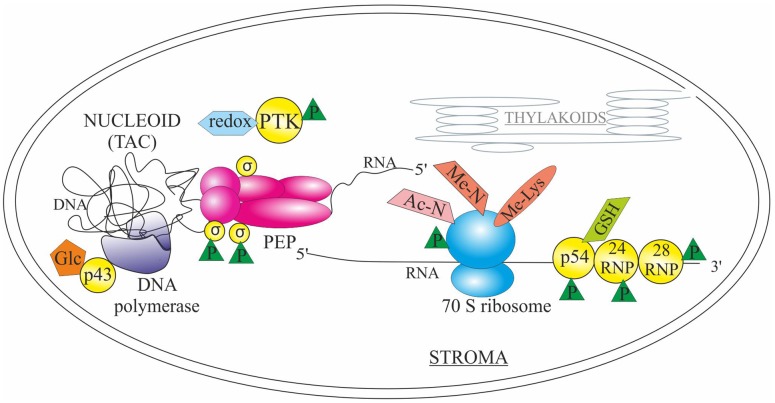
**Post-translational modifications in the regulation of plastid machinery for DNA replication and gene expression**. Glycosylation (Glc) of chloroplast protein p43 induces the activity of DNA polymerase. The sigma factors (σ) associated with the plastid encoded RNA polymerase PEP are regulated by (de)phosphorylation (P) conveyed by the plastid transcription kinase (PTK), which is autophosphorylated and redox-regulated. Glutathione-mediated redox regulation (GSH) and phosphorylation of endoribonuclease p54 affects the processing activity of *trnK* and *rps16* transcripts. Phosphorylation of RNA-binding proteins 24RNP and 28RNP affect the binding capacity to the 3′ end of chloroplast transcripts. RPL11 subunit of the 70S ribosome is trimethylated (Me-Lys), and ribosomes are also subjected to acetylation and monomethylation of N-terminal amino acid (Ac-N and Me-N, respectively), as well as phosphorylation. See text for details.

Organellar genomes are organized as nucleoids (also called as transcriptionally active chromosomes or TACs), DNA-protein complexes, which have been identified as the sites for both DNA replication and transcription (Melonek et al., 2016). Recently, proteomic analyses have suggested that also mRNA processing, splicing, editing, and ribosome assembly occur in association with the nucleoid, which supports the idea of co-transcriptional translation of plastid-encoded genes (Majeran et al., 2012). Although only few examples are thoroughly studied, PTMs of various types have been shown to regulate chloroplast genome replication and gene expression at multiple levels (**Figure 2**).

Even if a glycosylation machinery has been identified only in endoplasmic reticulum, some chloroplast proteins have been found to be glycosylated suggesting an existence of vesicular Toc/Tic independent chloroplast protein import route (Villarejo et al., 2005). One of the glycosylated proteins is the pea chloroplast protein p43, which associates with and activates the chloroplast DNA polymerase (Chen et al., 1996). Specifically, the N-terminal domain of p43 is highly O-arabinosylated (Gaikwad et al., 1999). Glycosylation of the protein is required for the induction of polymerization activity, although DNA binding is retained even if the protein is deglycosylated (Gaikwad et al., 1999, 2000). In addition to DNA replication, transcriptional activity of chloroplast genes is (partly) regulated by PTMs. Two different types of RNA polymerases, the plastid-encoded polymerase PEP, and the nuclear-encoded polymerase NEP, are responsible for the transcription of plastid-encoded genes (Shiina et al., 2005). The core subunits of PEP polymerase are associated with nuclear-encoded sigma factors, which are regulated by (de)phosphorylation (Link, 2003; Shimizu et al., 2010). Ser phosphorylation of the sigma factors (SIG6 being the most well studied one) is at least partly conveyed by the plastid transcription kinase (PTK), which is a chloroplast Ser/Thr protein kinase (Baginsky et al., 1997; Baena-González et al., 2001; Ogrzewalla et al., 2002; Salinas et al., 2006; Schweer et al., 2010). The kinase itself is regulated via autophosphorylation and glutathione-dependent redox regulation (Baginsky et al., 1997, 1999; Ogrzewalla et al., 2002). Effect of sigma factor phosphorylation on transcription depends on the sigma factor and the transcribed gene in question: for instance phosphorylation of the Thr_170_ in SIG1 inhibits transcription of the *psaA* gene (Shimizu et al., 2010), while phosphorylation of Ser_94/95_ and/or Ser_174_ in SIG6 enhances transcription of the *atpB* and *trnK* genes with no apparent effect on the transcription of the *psbA* gene (Schweer et al., 2010).

Processing of the chloroplast transcripts is also affected by phosphorylation and redox regulation of RNA binding proteins. For instance phosphorylation of endoribonuclease p54, which is responsible for the 3′ processing of the plastid *trnK* and *rps16*, affects the RNA processing activity but not the cleavage specificity (Nickelsen and Link, 1993; Liere and Link, 1994). Additionally, the processing activity of p54 was modulated by glutathione (Liere and Link, 1994). Phosphorylation of 24 kDa (24RNP) and 28 kDa (28RNP) RNA-binding proteins, associated with a complex regulating the maturation of the 3′ end of chloroplast transcripts (Hayes et al., 1996), has been shown to affect the affinity of the proteins to RNA. Specifically, phosphorylation of 24RNP increased its binding capacity to *petD* and *psbA* 3′ UTR (Loza-Tavera et al., 2006), whereas phosphorylation of 28RNP resulted in decreased affinity to RNA (Lisitsky and Schuster, 1995). Recently, it was shown that phosphorylation status of the 24RNP and 28RNP (and apparently other unidentified RNA binding proteins) mediates the interplay between the *petD* mRNA stability and processing (Vargas-Suarez et al., 2013).

The translational machinery of the chloroplast is composed of prokaryotic-type 70S ribosomes organized in small (Yamaguchi et al., 2000, 2003) and large (Yamaguchi and Subramanian, 2000) subunits. Chloroplast ribosomes contain rRNA and proteins, which are encoded both by the nuclear and chloroplast genomes (Carroll, 2013). Several ribosomal proteins in chloroplasts are targets of extensive PTMs, including formyl group or formyl methionine removal, N- and C-terminal processing, acetylation and monomethylation of N-terminal amino acids, trimethylation of Lys (Kamp et al., 1987; Schmidt et al., 1992; Yamaguchi and Subramanian, 2000; Yamaguchi et al., 2000; Alban et al., 2014) as well as phosphorylation (Guitton et al., 1984; Posno et al., 1984; Wagner et al., 2006). Recently, the enzyme responsible for the trimethylation of the internal Lys in Arabidopsis plastid ribosomal protein L11 (RPL11) has been identified as PrmA-like (Protein Arg methyltransferase-like) protein (Alban et al., 2014; Mazzoleni et al., 2015). Although depletion of Arabidopsis *PRMA-like* gene did not result in any phenotypic effects, mapping of the trimethylated Lys on the surface of the RPL11 protein allows hypothesizing that methylation might influence the stalk region, which is responsible for the recruitment of initiation, elongation and release factors (Mazzoleni et al., 2015).

A special case in the chloroplast gene expression processes is the regulation of *psbA* gene expression, which has been under intense study for decades. The *psbA* gene encodes the light-sensitive PSII core subunit D1, which is constantly degraded and resynthesized in a light-responsive PSII repair cycle (Aro et al., 1993; Mulo et al., 2008). It has been shown that in chloroplasts of green algae and higher plants *psbA* gene expression is mainly controlled at post-transcriptional levels (Mulo et al., 2012). In *Chlamydomonas reinhardtii*, ADP-dependent phosphorylation of the cPDI (chloroplast protein disulfide isomerase or RB60) protein in darkness leads to release of the protein from the 5′ UTR of *psbA* mRNA and cessation of translation (Danon and Mayfield, 1994). Additionally, binding of RB47 to the *psbA* mRNA is controlled via redox regulation of disulfide groups in RB60 (Danon and Mayfield, 1994; Alergand et al., 2006). It has also been hypothesized that phosphorylation of the spinach 28RNP (in addition to participating in 3′ UTR processing, see above) and ribosomal protein(s) might provide a light-dependent translation control mechanism for the chloroplast, especially during the repair cycle of PSII (Lisitsky and Schuster, 1995; Trebitsh et al., 2000; Yamaguchi and Subramanian, 2003).

## Light Reactions of Photosynthesis

**FIGURE 3:**
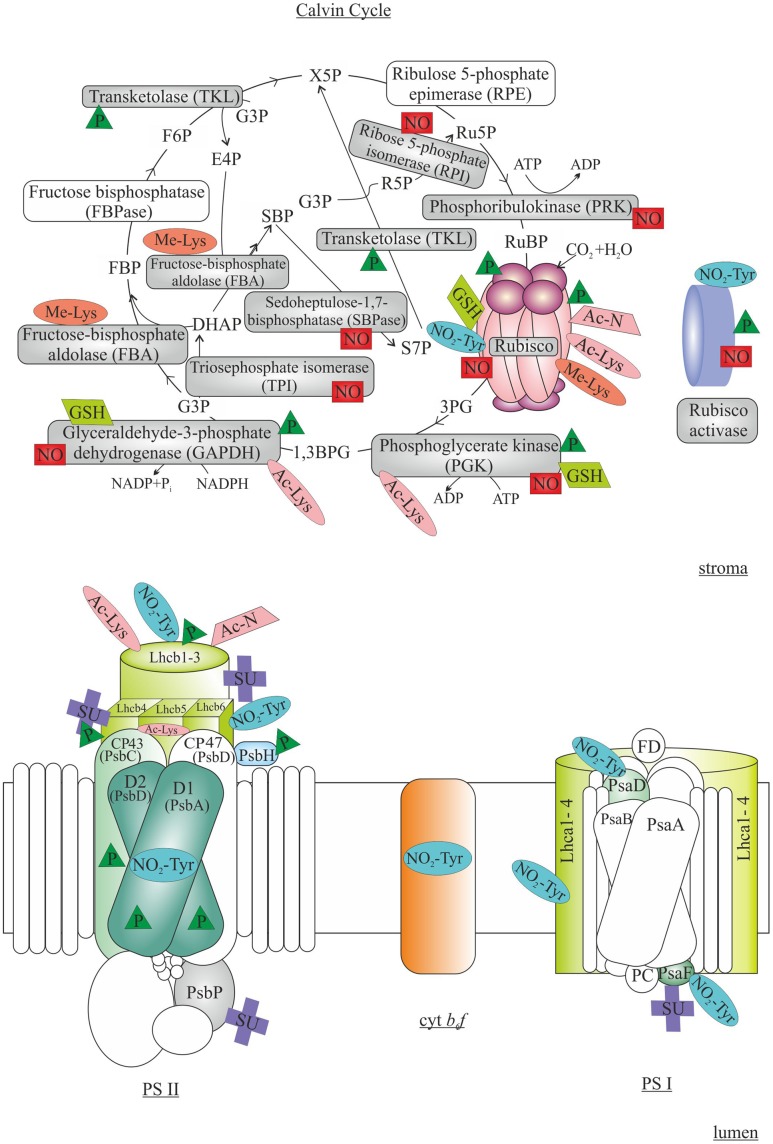
**Post-translational modifications in regulation of photosynthetic light reactions (Lower) and Calvin cycle (Upper)**. In light reactions, phosphorylation (P) of Photosystem (PS) II subunits D1, D2, CP43 and PsbH are involved in PSII repair cycle, while phosphorylation of light harvesting proteins (Lhcb) is required for state transitions. Tyr nitration (NO_2_-Tyr), N-terminal and Lys acetylation (Ac-N and Ac-Lys, respectively) and sumoylation (SU) of various PSII, Cytochrome b_6_f (Cyt b_6_f) and PSI subunits have been detected. In Calvin cycle, the function of Rubisco is controlled by a multitude of PTMs, including phosphorylation, Tyr-nitration, acetylation, Lys methylation (Me-Lys), nitrosylation (NO) and glutathionylation (GSH). Additionally, several other enzymes functioning in the Calvin cycle and activation of Rubisco are targets of various PTMs. The subchloroplastic sites of the PTMs are not indicated in the figure. 3PG, 3-phosphoglycerate; 1,3BPG, 1,3-bisphosphoglycerate; G3P, glyceraldehyde 3-phosphate; DHAP, dihydroxyacetone phosphate; FBP, fructose 1,6-bisphosphate; F6P, fructose 6-phosphate; X5P, xylulose 5-phosphate; R5P, ribose 5-phosphate; Ru5P, ribulose 5-phosphate; RuBP, ribulose 1,5-bisphosphate; S7P, sedoheptulose 7-phosphate; SBP, sedoheptulose 1,7-bisphosphate; E4P, erythrose 4-phosphate. The modified proteins are indicated with colors, while the non-modified proteins are shown as transparent. See text for details.

Light reactions of photosynthesis, i.e., capture of light energy by the light harvesting complex (LHC) for the production of reducing power (NADPH) occur at the thylakoid membrane via the thylakoid-embedded pigment-protein complexes, namely PSII, Cyt *b*_6_*f*, and PSI. Concomitantly, protons are pumped into the thylakoid lumen, and ADP is photophosphorylated to ATP upon release of the generated proton gradient via the ATP synthase (**Figure 3**). NADPH and ATP, in turn, are used for numerous reactions, carbon assimilation being the major process. PSII functions as an oxygen-plastoquinone oxidoreductase, which is prone to light-induced photoinhibition (Aro et al., 1993; Tyystjärvi, 2013). The PSII core proteins D1 and D2 as well as the inner antenna protein CP43 and a minor PSII subunit PsbH are targets for light-dependent Thr phosphorylation (**Figure 3**) catalyzed mainly by the STN8 kinase (Bellafiore et al., 2005; Bonardi et al., 2005; Fristedt and Vener, 2011), while the PSII CORE PHOSPHATASE is responsible for the reverse reaction (i.e., dephosphorylation; Samol et al., 2012). PSII protein phosphorylation is involved in the folding of the thylakoid membrane, which affects the lateral migration of damaged D1 protein from grana stacks to stroma lamellae for degradation and resynthesis (Tikkanen et al., 2008; Fristedt et al., 2009). Another well-studied phosphorylation process is involved in the balancing electron transfer between PSII and PSI according to ambient environmental cues (i.e., light quality and quantity). Phosphorylation of the light harvesting proteins Lhcb1, Lhcb2 and Lhcb4 is catalyzed by the STN7 kinase (Depege et al., 2003; Bellafiore et al., 2005), and instead of PSII, the phosphorylated LHC trimers deliver excitation energy to PSI (so called state transitions) to adjust the absorption cross sections of the two PSs (Rochaix, 2014). Dephosphorylation of LHC by the PPH1/TAP38 (chloroplast protein phosphatase/thylakoid associated phosphatase of 38 kDa) protein phosphatase, in turn, results in redistribution of excitation energy toward PSII (Pribil et al., 2010; Shapiguzov et al., 2010). The STN7 kinase is activated by the binding of plastoquinol to the Q_o_ site of Cyt *b*_6_*f* complex (Vener et al., 1997; Lemeille et al., 2009) and inhibited by stromal reductants (Rintamäki et al., 2000).

In addition to phosphorylation, LHC proteins are prone to various other PTMs (**Figure 3**), such as N-terminal acetylation (Michel et al., 1991; Wu et al., 2011; Rowland et al., 2015), Lys-acetylation (Finkemeier et al., 2011; Wu et al., 2011), Tyr-nitration (Galetskiy et al., 2011b) and sumoylation (Elrouby and Coupland, 2010; López-Torrejón et al., 2013). As acetylation neutralizes the positive charge either on the protein N-terminus or on Lys residue, it has numerous implications in biologic processes including determination of enzyme activity, protein stability and mediation of protein–protein interactions (Hwang et al., 2010; Bienvenut et al., 2011; Scott et al., 2011; Hoshiyasu et al., 2013). Accordingly, acetylation of the Lhcb1 and Lhcb2 proteins appear to be involved in the regulation of LHC attachment to the PSII complexes: the peripheral LHC antenna loosely bound to PSII showed higher level of Lys acetylation than the PSII-LHCII supercomplexes (Wu et al., 2011). In contrast to phosphorylation, acetylation status did not respond to changes in illumination (Wu et al., 2011). It is also worth noting that only the N-terminally trimmed form of Lhcb5 starting with Leu_38_ (other forms starting with Phe_39_ or Ser_40_) were reported to be Lys acetylated, indicating a cross-talk between N-terminal processing and acetylation of chloroplast proteins (Wu et al., 2011). Neither the chloroplast acetylation machinery (Dinh et al., 2015) nor the enzymes responsible for N-terminal processing (Rowland et al., 2015) have been thoroughly characterized yet. Also Tyr nitration of proteins representing PSII (including D1), Cyt *b*_6_*f*, PSI as well as LHC has been detected (Galetskiy et al., 2011a,b). Protein Tyr nitration is a marker of nitrosative stress, and it can irreversibly modify the conformation of proteins thus affecting the catalytic activity and susceptibility to proteolysis (Corpas et al., 2007). Indeed, changes in light conditions resulted in variation in nitration levels in different PSII-LHCII complexes, suggesting that nitration might be involved in photodamage, disassembly of complexes and subsequent degradation of proteins (Galetskiy et al., 2011a). It has also been found that LHC may be post-translationally modified by sumoylation (Elrouby and Coupland, 2010; López-Torrejón et al., 2013), which refers to covalent binding of the small ubiquitin-like modifier (SUMO) protein (Miura et al., 2007). Sumoylation has been implicated in the regulation of protein localization, interactions and catalytic activity (Vierstra, 2012). Obviously, the exact effects of these PTMs on the function of LHC require further studies.

## Carbon Assimilation and Starch Metabolism

The photosynthetic carbon reduction cycle, i.e., the Calvin cycle, is a multistep pathway in which redox equivalents and chemical energy (NADPH and ATP) originating from the light reactions is utilized for the reduction of atmospheric carbon dioxide into organic compounds. Calvin cycle involves 11 stromal enzymes, which catalyze 13 distinct reactions. In the first step, inorganic CO_2_ is fixed by ribulose-1,5-bisphosphate carboxylase/oxygenase (Rubisco), producing 3-phosphoglycerate (3PG), which is first phosphorylated and then reduced into glyceraldehyde-3-phosphate (G3P). G3P then exits from the Calvin cycle and is further used for the synthesis of more complex sugars, including starch that is the most abundant storage polyglucan in nature (Tetlow and Emes, 2014). Several Calvin cycle enzymes have been reported to be activated in light upon reduction of specific disulfide bonds by thioredoxin (Pedersen et al., 1966; Jensen and Bassham, 1968; Buchanan and Wolosiuk, 1976; Wolosiuk and Buchanan, 1976; Buchanan, 1980). In addition to redox regulation, all steps of CO_2_ fixation and starch metabolism are carefully controlled by multiple (PTM-dependent) mechanisms which balance the rate of starch synthesis with the availability of energy and carbon in different plant tissues and under various environmental conditions (**Figure 3**).

### Rubisco

In terrestrial plants and green alga, Rubisco exists as a holocomplex composed of eight nuclear-encoded small subunits (RBCS) and eight plastid-encoded large subunits (RBCL). Among the other enzymes involved in Calvin cycle Rubisco has been reported as a target of reversible phosphorylation in many plant species (**Figure 3**) (Reiland et al., 2009, 2011; Facette et al., 2013; Wang et al., 2014; Roitinger et al., 2015), RBCL being phosphorylated in response to light (Budde and Randall, 1990; Wang et al., 2014). The RBCL and RBCS subunits of Rubisco have been shown to contain multiple phosphorylation sites (Cao et al., 2011; Wang et al., 2014). Phosphorylation of the highly conserved RBCL residues Ser_208_, Thr_246_, Tyr_239_ and Thr_330_, located in the close proximity to RuBP binding site, might affect the catalytic activity of the enzyme (Lohrig et al., 2009; Hodges et al., 2013). Indeed, dephosphorylation of RBCL has been shown to result in decreased activity of the enzyme (Chen et al., 2011), perhaps via affecting the interaction between Rubisco and RA (Guitton and Mache, 1987; Aggarwal et al., 1993; Hodges et al., 2013). Moreover, it has been suggested that dephosphorylation of RBCL and/or RBCS may lead to dissociation of Rubisco holocomplex (Guitton and Mache, 1987; Aggarwal et al., 1993; Hodges et al., 2013).

Rubisco has also been found as a target of both N-terminal acetylation and Lys acetylation (**Figure 3**). In spinach, RBCL is post-translationally processed by removal of Met_1_ and Ser_2_ followed by the acetylation of the penultimate amino acid (Mulligan et al., 1988). Although N-acetylation of proteins in general is known to modify their activity and stability, the detailed significance and mechanism of RBCL N-termini modification remains unknown (Mulligan et al., 1988; Houtz et al., 1992; Zybailov et al., 2008). Lys acetylation of the Rubisco subunits has been identified only recently, and it has been reported as a dynamic modification in response to the changes in the energy status in plants under different light conditions (Gao et al., 2016). The Rubisco holocomplex contains multiple Lys acetylation sites (e.g., nine in Arabidopsis and thirteen in wheat; Finkemeier et al., 2011), which are localized either in the catalytic center of Rubisco (Cleland et al., 1998; Finkemeier et al., 2011), at the interface between the two RBCL subunits (Knight et al., 1990; Finkemeier et al., 2011) or at the site crucial for the formation of tertiary structure of Rubisco (Knight et al., 1990). Therefore, Lys-acetylation has been suspected to affect Rubisco activity and interactions between the subunits and with other molecules, and indeed recent studies have shown negative regulation of Rubisco activity by Lys acetylation (Finkemeier et al., 2011; Gao et al., 2016). Thus, acetylation of Rubisco might provide a mechanism to coordinate the function of light reactions and carbon assimilation with the carbon status of the cell.

In addition to acetylation, Lys residues of RBCL may be methylated (**Figure 3**). In many organisms (e.g., pea and tobacco), RBCL is considered as the main stromal methylprotein (Alban et al., 2014). Trimethylation of RBCL at Lys_14_ has been found in several plant species (Alban et al., 2014; Ma et al., 2016) as a modification catalyzed by the large subunit Rubisco methyltransferase (LSMT), a highly conserved SET-domain protein lysine methyltransferase found in all plant species (Dirk et al., 2006). Despite numerous studies, the role of Lys_14_ trimethylation of RBCL (as well as the role of methylation for other chloroplastic methylproteins) has not been identified (Clarke, 2013; Ma et al., 2016). Interestingly, in Arabidopsis, spinach, and wheat plants RBCL is not methylated at Lys_14_ indicating species-specific differences in regulatory mechanisms (Houtz et al., 1992; Mininno et al., 2012; Ma et al., 2016). The methylation of chloroplast proteins seems to be biologically important, as a mutant impaired in PTAC14 (plastid-located SET-domain methyltransferase) exhibits defects in chloroplast differentiation and shows an albino phenotype (Steiner et al., 2011). On the other hand, the LSMT knockdown plants do not show any decrease in CO_2_ assimilation and growth (Mininno et al., 2012).

Intriguingly, some Calvin cycle enzymes, including Rubisco, have been reported to be modified by peroxynitrite (**Figure 3**) (Cecconi et al., 2009; Lozano-Juste et al., 2011; Barroso et al., 2013). It has been suggested that Tyr-nitration of RBCL (and RA) might act as a modulator of plant defense-related responses including hypersensitive responses (Cecconi et al., 2009). On the other hand, Tyr-nitration of abundant proteins such as those involved in carbon metabolism might function as a non-specific scavenging system for reactive nitrogen forms under stress conditions. Reversibility of Tyr-nitration is still discussed, thus additionally raising new questions about a potential function as a specific signaling event (Souza et al., 2008; Baudouin, 2011).

The reversible S-nitrosylation of Rubisco Cys residues has been reported both *in vitro* and *in vivo* for several plant species in response to nitric oxide (NO) -releasing compounds or to abiotic stresses (**Figure 3**) (Abat et al., 2008; Abat and Deswal, 2009; Fares et al., 2011; Vanzo et al., 2016). As the redox-active thiols in Cys residues can be modified by the covalent binding of NO resulting in the formation of S-nitrosothiol (Lindermayr et al., 2005), it is plausible that S-nitrosylation of Cys residues adjacent to the Rubisco active site in Arabidopsis might regulate the activity of the enzyme and degradation of the protein (Takahashi and Yamasaki, 2002; Marcus et al., 2003; Romero-Puertas et al., 2008). Indeed, recent enzymatic activity assays have revealed that Rubisco inactivation in response to S-nitrosylation is probably the main cause of reduction in carbon fixation upon various stress conditions (Clark et al., 2000; Abat et al., 2008; Abat and Deswal, 2009).

Another modification of Cys residues is protein S-glutathionylation, a well-described mechanism of signal transduction and protein regulation in mammals (Chrestensen et al., 2000). S-glutathionylation is a reversible post-translational formation of a mixed disulfide between the Cys residue of protein and glutathione. Previously, three Cys residues in RBCL and one in RBCS have been identified as targets of S-glutathionylation in plants (Rouhier et al., 2005), green alga (Zaffagnini et al., 2012a) and cyanobacteria (Sakr et al., 2013; Chardonnet et al., 2015). Protein S-glutathionylation probably protects specific Cys residues against irreversible oxidation under stress conditions (Ito et al., 2003; Zaffagnini et al., 2012b), but this PTM can also result in modulation of protein activity (Klatt and Lamas, 2000; Fratelli et al., 2004) and localization (Chardonnet et al., 2015). Nevertheless, the functional significance of Rubisco S-glutathionylation is not known yet.

### Activation and Function of the Calvin Cycle

Although PTMs of Rubisco have been extensively studied, also numerous other enzymes involved in carbon assimilation have been shown to possess multiple PTMs (**Figure 3**). As RA is responsible for removing inhibitors from Rubisco active center and thus contributes to initiation of carbon fixation, the stimuli affecting the RA activity is reflected in the yield of the entire carbon assimilation cycle. In green alga *C. reinhardtii*, RA is phosphorylated at Ser_53_ by the thylakoid-localized Stn7 ortholog Stt7 kinase (see above; Lemeille et al., 2010). RA is mainly localized in the stroma, but a smaller portion of the enzyme has been found in association with the thylakoid membrane (Jin et al., 2006). It has been suggested that phosphorylation of RA increases the attachment of RA to the membrane, protecting Stt7 against proteolysis (Lemeille et al., 2009, 2010). The relocation could also be a mechanism reducing the activity of Rubisco under specific environmental conditions (Lemeille et al., 2010). In Arabidopsis plants, RA is phosphorylated at two sites, Thr_78_ and Ser_172_ (Boex-Fontvieille et al., 2014). In the dark, the phosphorylation percentage of Thr_78_ increases (Reiland et al., 2009; Kim et al., 2016). As Thr_78_ is located in the region crucial for Rubisco interaction (Zhang and Portis, 1999; Kim et al., 2016), it has been suggested that Thr_78_ phosphorylation inhibits Rubisco activation (van de Loo and Salvucci, 1996; Stotz et al., 2011; Boex-Fontvieille et al., 2014). However, the importance of Thr_78_ phosphorylation for the Rubisco activation requires further investigation as the Thr_78_ is not conserved and replaced by Ile in maize and rice (Baginsky, 2016).

In addition to Rubisco, three other enzymes involved in Calvin cycle have been reported as phosphoproteins. Phosphoglycerate kinase (PGK) enzyme catalyzing the transfer of phosphate group from ATP to 3PG is phosphorylated in Arabidopsis, rice, and maize plants (Reiland et al., 2009; Facette et al., 2013; Roitinger et al., 2015; Baginsky, 2016). The two latter species share the identical phosphorylation site VGAVSpSPK whereas in Arabidopsis PGK is phosphorylated in a domain much closer to the N-terminus. The kinase responsible for phosphorylation is unknown, but the phosphorylation motif suggests proline-directed kinase as a possible candidate (Baginsky, 2016). Glyceraldehyde 3-phosphate dehydrogenase (GAPDH) possesses several phosphorylation sites, but as the sites differ significantly between different organisms, it is plausible that phosphorylation is not a major determinant of GAPDH activity in chloroplasts (Baginsky, 2016). Moreover, the main transketolase isoform in Arabidopsis (TKL1) is phosphorylated in a Ca^2+^ dependent manner at Ser_428_, and phosphorylation affects enzyme activity (Rocha et al., 2014). Although Ser_428_ is conserved in higher plants, Ser_428_ has been found phosphorylated only in Arabidopsis plants (Hou et al., 2015; Baginsky, 2016). It is intriguing that PGK and GAPDH are also targets of Lys acetylation and S-glutathionylation (Finkemeier et al., 2011; Zaffagnini et al., 2012a; Chardonnet et al., 2015; Shen et al., 2015). The enzymatic activity of GAPDH and PGK is increased upon deacetylation, but the functional importance of S-glutathionylation of GAPDH and PGK remains to be elucidated (Finkemeier et al., 2011; Shen et al., 2015). These examples indicate that further studies are urgently needed in order to fully understand the dynamic regulation of Calvin cycle enzymes and to pinpoint the responsible enzymes involved (Friso and van Wijk, 2015; Baginsky, 2016).

Furthermore, a number of other enzymes involved in carbon assimilation have been shown to be post-translationally modified. For instance, fructose 1,6-bisphosphate aldolase (FBA) is trimethylated at a conserved Lys residue close to the C-terminus of the protein, however, without any effect on catalytic activity or the oligomeric state of the enzyme (Mininno et al., 2012; Ma et al., 2016). In poplar trees sedoheptulose-bisphosphatase (SBPase), RA, ribose-5-phosphate isomerase (RPI), phosphoribulokinase (PRK), GAPDH, triosephosphate isomerase (TPI), and PGK were S-nitrosylated during short-term oxidative stress induced by NO treatment (Vanzo et al., 2014, 2016), but the functional importance has not been described yet (Lindermayr et al., 2005; Abat et al., 2008; Romero-Puertas et al., 2008; Abat and Deswal, 2009).

### Starch Metabolism

Starch synthesis and degradation occur in a coordinated manner on a diurnal basis. In leaves, starch is synthesized during the day and degraded in darkness (Kötting et al., 2010). Reversible protein phosphorylation plays an important role also in the regulation of starch metabolism (Tetlow et al., 2004a, 2008; Grimaud et al., 2008; Reiland et al., 2009), and five different phosphoproteins (phosphoglucose isomerase, phosphoglucomutase, starch synthase and two subunits of ADP-glucose pyrophosphorylase) involved in starch biosynthesis have been identified in Arabidopsis leaves (Geigenberger, 2011). Interestingly, starch synthase has been reported to be phosphorylated in a light dependent manner, i.e., exclusively at the end of the dark period (Reiland et al., 2009). Analyses of amyloplasts and chloroplasts from *Triticum aestivum* (wheat) have shown that some isoforms of starch-branching enzymes (SBE) are catalytically activated by phosphorylation and deactivated by dephosphorylation of one or more of their Ser residues (Tetlow et al., 2004b). Additionally, phosphorylation is apparently involved in the formation of protein complexes composed of starch synthase, SBE isoforms as well as other enzymes with undefined role(s) (Tetlow et al., 2004b; Kötting et al., 2010). It has been speculated that the physical association of the enzymes could alter their activities thus improving the efficiency of starch polymer construction (Kötting et al., 2010; Geigenberger, 2011). Moreover, numerous enzymes involved in starch metabolism, such as glucan water dikinase (GWD, also termed SEX1), starch excess4 (SEX4), β-amylase 1 (BAM1), ADP-glucose pyrophosphorylase, ADP-Glc transporter and class II SBE (Mikkelsen et al., 2005; Balmer et al., 2006; Sokolov et al., 2006; Valerio et al., 2011; Tuncel et al., 2014) are redox activated by thioredoxin. However, it is worth noting that redox modification of starch biosynthesis enzymes in response to light (and other environmental stimuli; reviewed in Kötting et al., 2010; Geigenberger, 2011) is not the only determinant of starch accumulation in plants, but most probably other (PTM-dependent) regulatory mechanisms will be identified in the future (Li et al., 2012).

## Conclusion

Recently developed new experimental tools, i.e., PTM-specific antibodies and stains as well as enrichment techniques and high quality equipment for mass spectrometry have enabled identification of a range of PTMs in chloroplast proteins. Detailed knowledge about the effects of protein phosphorylation and redox regulation on the photosynthetic reactions already exists, but the regulation of most metabolic pathways in the chloroplast is poorly understood. Because a specific amino acid residue may be targeted by different PTM types (e.g., Lys methylation or Lys acetylation), and because different PTMs may have either antagonistic or cooperative effects, it will be important to reveal the entire PTM code of a protein(s) in order to understand the physiological significance of PTM-mediated regulation in a given metabolic pathway. Future studies are likely to reveal novel modification types as well as molecular mechanisms of PTM-dependent regulation of various metabolic pathways in chloroplasts.

## Author Contributions

PM, MG, and MK have made substantial intellectual contribution to the work, participated in writing and revised the paper. MK and MG have drawn the figures. All authors have approved the paper for publication.

## Conflict of Interest Statement

The authors declare that the research was conducted in the absence of any commercial or financial relationships that could be construed as a potential conflict of interest.

## Abbreviations

PS: photosystem
*psa*: genes encoding subunits of Photosystem II
*psb*: genes encoding subunits of Photosystem II
PTM: post-translational modification
RA: Rubisco activase
RB: RNA binding protein
*rps*: genes encoding ribosome subunits
*trn*: genes encoding chloroplast transferRNAs
